# Integrating Scalable Genome Sequencing Into Microbiology Laboratories for Routine Antimicrobial Resistance Surveillance

**DOI:** 10.1093/cid/ciab796

**Published:** 2021-11-25

**Authors:** Mihir Kekre, Stefany Alejandra Arevalo, María Fernanda Valencia, Marietta L Lagrada, Polle Krystle V Macaranas, Geetha Nagaraj, Anderson O Oaikhena, Agnettah M Olorosa, David M Aanensen, NIHR Global Health Research Unit on Genomic Surveillance of Antimicrobial Resistance, Khalil Abudahab, Monica Abrudan, Silvia Argimón, Harry Harste, Dawn Muddyman, Ben Taylor, Anthony Underwood, Nicole Wheeler, Sophia David, Pilar Donado-Godoy, Johan Fabian Bernal, Alejandra Arevalo, Erik C D Osma Castro, K L Ravikumar, Varun Shamanna, Vandana Govindan, Akshata Prabhu, D Sravani, M R Shincy, Steffimole Rose, K N Ravishankar, Iruka N Okeke, Ayorinde O Afolayan, Jolaade J Ajiboye, Erkison Ewomazino Odih, Celia Carlos, June M Gayeta, Elmer M Herrera, Ali Molloy, John Stelling, Carolin Vegvari

**Affiliations:** 1 Centre for Genomic Pathogen Surveillance, Big Data Institute, University of Oxford, Old Road Campus, Oxford, United Kingdom; 2 Wellcome Genome Campus, Hinxton, United Kingdom; 3 Colombian Integrated Program for Antimicrobial Resistance Surveillance—Coipars, CI Tibaitatá, Corporación Colombiana de Investigación Agropecuaria (AGROSAVIA), Tibaitatá–Mosquera, Cundinamarca, Colombia; 4 Research Institute for Tropical Medicine, Muntinlupa, Philippines; 5 Central Research Laboratory, Kempegowda Institute of Medical Sciences, Bengaluru, India; 6 Department of Pharmaceutical Microbiology, University of Ibadan, Ibadan, Nigeria

**Keywords:** whole-genome sequencing, WGS, microbiology laboratory, antimicrobial resistance, AMR surveillance

## Abstract

Antimicrobial resistance (AMR) is considered a global threat, and novel drug discovery needs to be complemented with systematic and standardized epidemiological surveillance. Surveillance data are currently generated using phenotypic characterization. However, due to poor scalability, this approach does little for true epidemiological investigations. There is a strong case for whole-genome sequencing (WGS) to enhance the phenotypic data. To establish global AMR surveillance using WGS, we developed a laboratory implementation approach that we applied within the NIHR Global Health Research Unit (GHRU) on Genomic Surveillance of Antimicrobial Resistance. In this paper, we outline the laboratory implementation at 4 units: Colombia, India, Nigeria, and the Philippines. The journey to embedding WGS capacity was split into 4 phases: Assessment, Assembly, Optimization, and Reassessment. We show that on-boarding WGS capabilities can greatly enhance the real-time processing power within regional and national AMR surveillance initiatives, despite the high initial investment in laboratory infrastructure and maintenance. Countries looking to introduce WGS as a surveillance tool could begin by sequencing select Global Antimicrobial Resistance Surveillance System (GLASS) priority pathogens that can demonstrate the standardization and impact genome sequencing has in tackling AMR.

The World Health Assembly’s Global Action Plan recognized antimicrobial resistance (AMR) as a multifactorial global threat [1–4]. Novel drug target discovery is lagging and needs to be complemented with systematic and standardized epidemiological surveillance. This strategy could potentially have a strong influence on evaluating the effectiveness of existing treatments, strengthening epidemiological modeling to identify outbreaks and their high-risk clonal lineages, and incorporating evidence-based changes to regional and national policies tackling the spread of AMR.

Currently, surveillance data in the World Health Organization’s (WHO’s) Global Antimicrobial Resistance Surveillance System (GLASS) are generated via the characterization of phenotypic responses of bacteria on certain growth media, and in the presence of antimicrobial agents. These tests, regarded as the “gold standard,” help identify the strains and determine their pathogenic potential and antimicrobial susceptibility profile (ASP). The knowledge gained is then utilized by a network of clinicians, microbiology laboratories, and public health bodies to revise individual patient treatment and policy. It is widely accepted that high-quality antimicrobial susceptibility profiling forms the cornerstone of a strong AMR surveillance program, but this gold-standard approach can have certain limitations, such as (1) poor scalability for true epidemiological investigations, (2) labor-intensive and extended times-to-result, and in some cases, (3) scarcity of domain expertise [4–7]. Operationally, the separate, distinct laboratory workflows used to characterize bacterial species can also lengthen the time-to-result.

In more recent times, molecular assays have been introduced to supplement traditional typing methods. From cultured bacterial colonies, molecular AMR tests allow the following: (1) faster typing (of slow-growing bacteria); (2) verification of known mechanisms of drug resistance; (3) flexibility with cost, laboratory investment, and expertise; and (4) higher sample sensitivity and accuracy of results [6, 8]. There is a strong case for whole-genome sequencing (WGS) to provide enhancements to the phenotypic data.

WGS allows the simultaneous screening of all referenced AMR loci and genotypic signatures in the DNA of an isolate through a single sequencing run following microbial culture [4, 7, 9]. Once sequenced (to a coverage depth of at least 25×), these genomes can be reanalyzed retrospectively and repeatedly for new markers of resistance, as and when novel ones are identified. WGS-based surveillance has the potential to provide the highest possible resolution with rapid identification of outbreaks and high-risk clonal transmission events. It is crucial to point out that, for clinical treatment, WGS is currently best utilized in conjunction with phenotypic reporting. WGS cannot quantifiably ascertain AMR and also requires predetermined species and ASP indications [4]. However, WGS could verify discordant ASPs, and for some organisms it could be the stand-alone assay for surveillance. WGS is becoming increasingly rapid and affordable for surveillance (albeit following an initial capital investment), due to the increased output of current-generation sequencers that allow multiple pathogen genomes to be sequenced in parallel [10].

For real-time surveillance to be realized globally, microbial WGS data need to be analyzed across time and space, to identify outbreaks and hypothesize geographic transmission events by comparing the relatedness of each sampled strain against others in the demographic. There have been instances of well-established surveillance systems, but these have been limited to high-income settings. Low- and middle-income countries (LMICs) often lack comprehensive monitoring, largely due to challenges in laboratory network funding and standardized data reporting [5].

To establish global AMR surveillance using WGS, we developed a laboratory implementation approach that we applied within the NIHR (National Institute for Health Research) Global Health Research Unit (GHRU) on Genomic Surveillance of Antimicrobial Resistance, a partnership of national and regional reference laboratories, academic centers, and private organizations.

## WGS IMPLEMENTATION JOURNEY

**Figure 1.**
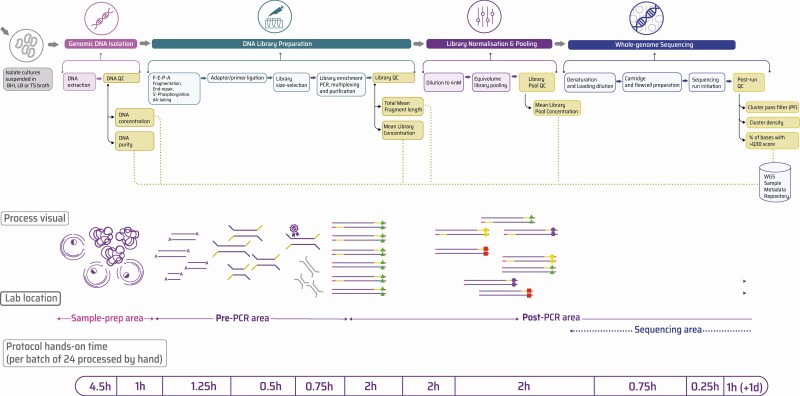
A typical laboratory workflow to perform WGS starting with pure bacterial colony isolates. Isolates were grown in Sigma Aldrich’s BHI, lysogeny or TS broths, followed by genomic DNA isolation and quantification (using ThermoFisher Nanodrop for nucleic acid purity and ThermoFisher Qubit for absolute DNA concentration). Next, double-stranded libraries were constructed using a combination of fragmentation, adaptor ligation, and the addition of multiplexing oligos (or oligo barcodes). Finally, these libraries were sequenced on an Illumina (MiSeq) sequencer using SBS chemistry. To access the full list of SOPs for this workflow, see https://www.pathogensurveillance.net/resources/protocols/ [19]. Abbreviations: BHI, brain-heart infusion medium; PCR, polymerase chain reaction; QC, quality control; SBS, sequencing-by-synthesis; SOP, standard operating procedure; TS, tryptone soy; WGS, whole-genome sequencing; PF, pass filter.

A prerequisite to enhancing AMR surveillance with WGS is a robust phenotypic testing setup. Four units—in Colombia, India, Nigeria, and the Philippines—were experienced with conventional bacterial typing and had rigorous protocols in place. This included an operational setup that verified the identity of all organisms referred from collection sites, testing their susceptibility against a panel of antimicrobials, and detecting previously published virulence factors [5, 11]. No functional assays of virulence were performed. The resulting sample metadata were collected and integrated [12]. Figure 1 illustrates a typical WGS laboratory workflow when implemented downstream of phenotypic testing.

**Figure 2.**
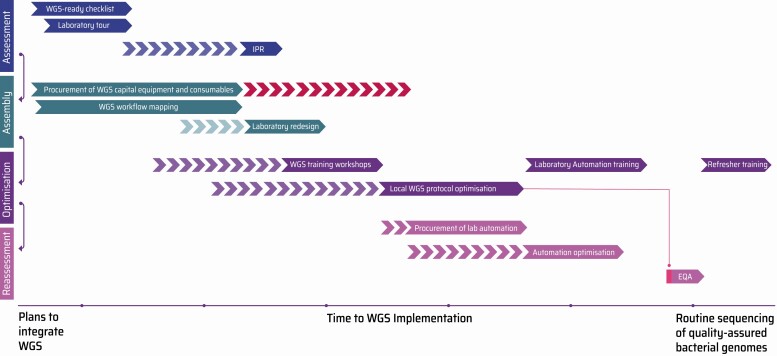
The WGS Implementation Journey (WIJ) Roadmap. A typical WIJ consisted of 4 distinct phases of activities—(1) Assessment: precise review of laboratory infrastructure using the WGS-ready checklist (Supplementary Information) followed by an IPR to evaluate competency in molecular methods (Supplementary Figure 1); Assembly: workflow mapping, procurement of WGS equipment and consumables (Supplementary Table 1), followed by a laboratory redesign if needed (Supplementary Figure 2) (based on whether a laboratory has the necessary equipment, the IPR can be fast-tracked); Optimization: hands-on WGS training workshops organized by experienced instructors; and Reassessment: automation and quality assurance of DNA, library preparation, and genome sequencing methods, making them more scalable, cost-effective, and sustainable. Subsequently, an EQA can be performed as an independent verification of process quality. As shown, EQA audits should not be fast-tracked and only be performed once laboratory users have had sufficient time to demonstrate proficiency at DNA preparation, library creation, and sequencing protocols. In the event that a participating laboratory does not pass an EQA, refresher training (or re-optimization) could be offered before the next assessment. Abbreviations: EQA, External Quality Assessment; IPR, Internal Process Review; WGS, whole-genome sequencing.

Embedding quality-assured genome sequencing requires careful planning, long before the purchase of a sequencer. In addition to the initial investment in equipment and reagents, there are crucial challenges and considerations for any new laboratory to ensure reliable, cost-effective, and reproducible quality of genomes. A pragmatic roadmap for prospective laboratories looking to assemble WGS processing power has been compiled, based on our experience (Figure 2).

### WGS Implementation Journey Roadmap

Typically, laboratories with finite resources and budgetary constraints, and therefore diminished purchasing power, prioritize low assay costs and ease of sample processing over other factors. Bacterial WGS supports this, as it (1) simplifies laboratory workflows by utilizing the same protocol across all organisms of interest; (2) drastically decreases time-to-result, especially against lengthy *Salmonella* serotyping assays and for slow-growing bacteria like *Mycobacterium tuberculosis*; and (3) benefits from extensive multiplexing opportunities that can help sequence several bacterial genomes on a single run [6, 13–15]. A general prerequisite to rapidly setting up WGS is the verified ability to perform high-quality polymerase chain reaction (PCR), since preparing WGS libraries involves similar techniques.

The journey to embedding WGS capacity can be split into 4 phases of activity: Assessment, Assembly, Optimization, and Reassessment.

#### Assessment

##### Considerations.

The assessment phase serves to evaluate existing infrastructure for the potential to expand routine phenotypic AMR typing to WGS. Additionally, it allows for extensive engagement with laboratory scientists and microbiologists on-site to understand their existing laboratory-based workflows and proficiencies.

##### Challenges.

First, the equipment, footprint, and safety guidelines required for routine microbiology differ greatly from those needed to run WGS. Like any molecular method that utilizes DNA as starting material, genome sequencing is performed on benches separate to bacterial culture. In reality, this cannot always be achieved at nascent laboratories due to extremely limited space or workbenches being shared among several research groups. Second, in our case, performing individual on-site assessments was not always practical because the partner locations were located around the world.

##### Solutions.

Laboratories were presented with a checklist questionnaire designed in-house to evaluate WGS setup potential. Its purpose was to rapidly examine the premises and personnel skills, and to provide recommendations on infrastructure reconfiguration (ie, dedicated workspaces, sterility measures, and controlled environments in which to perform sequencing). Laboratories were free to flexibly modify these suggestions based on their situation. Our partner sites in Colombia, Nigeria, and the Philippines reorganized or extended their laboratory footprint, while our partner site in India remodeled from the ground up. A representative WGS-ready checklist is available (Supplementary Information).

Based on the checklist responses, our partners undertook an Internal Process Review (IPR) to identify potential gaps in practical laboratory expertise. This exercise verified each laboratory’s familiarity with DNA isolation and quantification, PCR amplification, and sample transport between sites (all necessities upstream to sequencing). Supplementary Figure 1 illustrates the IPR exercise.

#### Assembly

##### Considerations.

WGS is characterized by specialized capital equipment and consumables and hence reinforces the need to build strong relationships with local suppliers to run a sustainable operation. The assembly phase leans heavily on experimental planning, and the procurement of equipment Capital Expenditures (Capex) and reagents/labware Operational Expenses (Opex). The aim with the GHRU on Genomic Surveillance of Antimicrobial Resistance initiative was to assemble a cost-effective short-read WGS pipeline that could rapidly and routinely sequence genomes from gram-positive and -negative bacterial species to a minimum coverage depth of 25× per nucleotide base position.

##### Challenges.

Laboratories on-boarding WGS for AMR surveillance need to carefully consider the affordability of both Capex and the recurring Opex. Emerging WGS territories are serviced by local distributors of the parent provider (sometimes called subsidiaries or channel partners). Here, product pricing is entirely demand-driven, poorly regulated, heavily taxed upon import, and controlled almost exclusively by the local distributor. This leads to highly inflated device and reagent costs [6]. Due to lower demand in our partner countries, we observed that distributors never manufactured locally and were often forced to deliver perishable stock that is closer to expiry. Fresh reagent stocks were only imported periodically with shipping delays, and usually had a longer turnaround time that, in turn, required laboratory managers to purchase months in advance. Paradoxically, we also found that LMIC laboratories such as our partner units spent significantly more on Capex and Opex items than their counterparts in established next-generation sequencing (NGS) markets did. Additionally, after-sales support from equipment/reagent suppliers does not meet the expected level of standard, with severe delays in customer service and a lack of promptness and accuracy with technical troubleshooting.

Moreover, there are several sequencing platform types available to perform microbial genomics. Selecting the optimal technology depends on factors like eventual application, current and expected sample turnover, sequence read lengths, required depth of coverage, time to result, and the acceptable assay running cost.

##### Solutions.

Mapping out essential Capex and Opex helps address process gaps and negotiate fit-for-purpose products from vendors. To accelerate time-to-purchase and instrument validation, we compiled a WGS Lab Toolkit of the main equipment, labware, and reagents that partner laboratories could use as a guide to navigate the plethora of procurement options (Supplementary Table 1). It is worth noting that this list represents just one of several ways to assemble a functional WGS suite—one that was well suited to our budget and applications.

Preferred suppliers (specially in LMICs) were chosen based on the following criteria:

1. Technology relevance (best-in-class or equivalent)
2. Ease of access, convenient format for routine use
3. Previous validation on bacterial isolates or bacterial WGS protocols
4. Low operational assay costs and maintenance premiums (if applicable)
5. Global presence with reliable supply chains and delivery dispatch networks
6. Swift, efficient technical support and issue escalation that is regionally available

Using the WGS-ready checklist, transformational changes were enforced upon the arrival of equipment. Supplementary Figure 2 illustrates a representative reconfiguration of a laboratory to accommodate WGS alongside microbiology.

#### Optimization

##### Considerations.

This phase involved refining laboratory protocols that would enable WGS of collected bacterial isolates (Figure 1). This consisted of the following: (1) validation of instruments; (2) hands-on training in DNA isolation and quantification (if needed), library preparation and quality control (QC), initiation and assessment of a short-read Illumina sequencing run; and (3) development of standard operating procedures (SOPs) or protocols for routine use.

##### Challenges.

Practical WGS training courses offered online, at a university or as part of joint high-income-country–LMIC research initiatives, are generally organized within typical, nonchallenging environments. For scientists in LMICs, these are almost always organized abroad and cost a significant amount to attend. Most workshops go into great depth about specific techniques, yet they ignore key aspects in laboratory management. Methods are taught using an “ideal recipe sequence” style that does not prepare beginners for real-world scenarios. We also therefore found that the newly acquired skills could not easily be transferred to one’s home laboratory upon return due to a different environment and work dynamic. Moreover, courses organized on-site offered little value if instructors did not fully understand local logistical nuances and complexities before initiating training.

Generally, scientific journals do not enable authors to provide a detailed list of protocols alongside scientific work, which can make it challenging for users to replicate or introduce adjustments to published work. Additionally, we observed that a lack of standardized annotation, formulation, and archiving of SOPs led to poor reproducibility of results, both between laboratories and between operators within a laboratory.

##### Solutions.

Once the equipment was fully validated, local on-site training could commence. Fully integrated workshops were delivered free-of-charge at the partner’s laboratory site within the exact working environment and layout required. Hands-on coordination was needed to ensure the advanced preparation and timely arrival of the appropriate reagents, labware, and course handbooks. The instructor–trainee ratio was maintained at a maximum of 1:3 to encourage healthy levels of interaction and engagement. This also ensured that the instructor was able to stock each participant’s workstation adequately. Hands-on “recipe-style” protocol repetition was blended with interactive discussions and coursework on the principles of WGS, current technology, and applications in clinical microbiology. This empowered participants with no prior sequencing experience to understand the benefits, limitations, and scalability of laboratory genomics in a public health context. Alongside technical competence, we trained team managers in project planning, laboratory biosafety, quality assurance, procurement, supply chain, SOP compilation, and sample data management.

The Train-the-Trainer model addressed the scalability of laboratory training through improved onward training coverage, thereby reducing operational costs [16, 17]. The aim was to work with a cohort of trainees knowledgeable about regional challenges to build technical proficiency in AMR and WGS techniques, and pedagogical skills to efficiently share expertise with neighboring or collaborating staff and researchers. The overall goal was to ensure organic growth of a network of regional trainers who will teach, mentor, and share lessons with further audiences locally.

To enhance the reproducibility and consistency of results across sites, a consolidated catalog of optimized laboratory SOPs to perform bacterial WGS was made available on protocols.io [18, 19]. The effectiveness of SOPs, especially when utilized across independent sites within a surveillance network, is highly dependent on monitoring DNA/library quality and sequencing run performance (described as QC checkpoints in Figure 1). Every stage of the WGS workflow must also be supplemented with suitable positive and negative assay controls in order to facilitate quality assurance (QA)—for example, pre-quantified and pre-speciated DNA libraries as positive controls within library QC or quality-controlled PhiX libraries spiked into a sequencing run [19].

#### Reassessment

##### Considerations.

Reviews were initiated 6–9 months after end-to-end WGS laboratory expertise was delivered. This allowed each laboratory to retrain, practice, and optimize methods taught during the optimization phase. Reassessment served to review and improve throughput of sample processing to meet any increase in demand, and to evaluate batch-to-batch genomic data quality, which, in turn, indicated the degree of training success.

##### Challenges.

First, when the demand for routine WGS grows, manual DNA and library preparation becomes increasingly expensive, time-consuming, and error-prone. Currently, staff are limited to processing smaller batches due to restrictions imposed by laboratory equipment or benchtop sample degradation.

Second, QA schemes are carried out extensively for microbiological laboratories that wish to challenge and accredit their protocols [11]. Previous attempts to recreate this for WGS have failed due to lack of resources and globally recognized QC standards for genomic data [20]. This is further complicated by the need to tightly control DNA extraction outputs, library preparation methods, and sequencing chemistry within a preferred platform.

##### Solutions.

To address the first challenge, liquid-handling robotics help realize more efficient sample processing, while reducing consumable costs and turnaround times [21, 22]. It enables the processing of batch sizes approximately 2–3-fold larger in the same time frame as protocols done by hand, while drastically improving batch to batch quality [21, 22]. A reassessment exercise often helps laboratories transform manual SOPs into automated workflows. Besides pipetting accuracy and versatility, we found that sustainable liquid handlers that addressed the challenges at resource-limited laboratories shared the following salient features: (1) affordably priced hardware (with fixed pricing for most global regions); (2) easily installable hardware and software setup; (3) protocol development included within instrument cost; (4) flexible, drag-and-drop application programming interface for method development; (5) online repository of prevalidated methods scripts for DNA extraction, DNA quantification, PCR master mix preparation, WGS library construction, and DNA and library cherry-pick/dilution; (6) self-repairable hardware and virtual service support (where possible); (7) reagent/kit agnostic liquid dispensing; and (8) compatibility with low-cost, nonproprietary (generic) labware on deck.

To address the second challenge, we piloted an external QA exercise for bacterial whole genomes, which provided an independent verification of laboratory standards with a panel of blinded, phenotypically and genotypically well-characterized test isolates. This will be useful as WGS becomes more widely used for AMR surveillance. It is important that such QA schemes include traditional phenotypic identification/antimicrobial susceptibility testing alongside wet/dry laboratory pathogen genome sequencing, to help maintain backwards compatibility between genomic results and traditional “gold standard” counterparts [4, 13, 14]. For wet laboratories, this includes reporting phenotypic AMR, quality metrics for DNA and library preparation, and run performance metrics like run yield (in Gb), cluster density, cluster pass filters and percentage of bases with quality scores greater than Q30.

### WGS Implementation Journey Vignettes and Experiences With GHRU

A catalog of laboratory setup challenges and solutions implemented as part of the GHRU project in Colombia, India, Nigeria, and the Philippines are documented in Supplementary Table 2.

## BUILDING SUSTAINABLE WGS LABORATORY NETWORKS

Based on the challenges discussed, there are some important long-term considerations when assembling an impactful laboratory network capable of sequencing bacterial genomes sustainably.

### Network Design

Central or national reference laboratories (where WGS will generally first be implemented) have a network of regional and sentinel laboratories and collection sites, each of which coordinates sampling and reporting within their demographic, and feeds data upwards into the network coordinated by the wider authority. Outbreak control for the 2019 severe acute respiratory syndrome coronavirus 2 (SARS-CoV-2) pandemic has brought about vast investment in PCR-based testing, even at remote pop-up locations; this bodes well for peripheral sites aiming to up-skill in processes upstream to sequencing.

**Figure 3.**
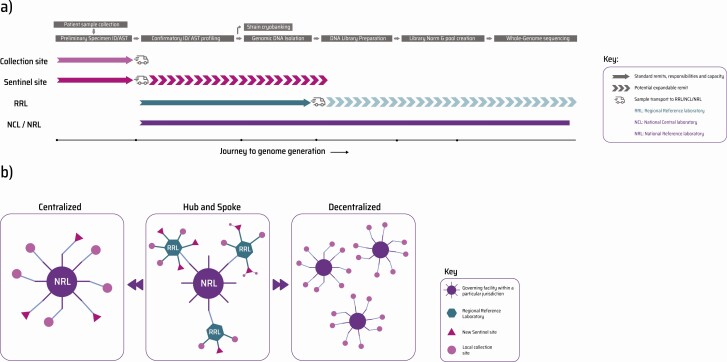
Scalability of laboratory networks within a WGS-based AMR surveillance system. (*a*) Surveillance networks implementing AMR action plans consist of 4 broad tiers (with varying nomenclature globally). Collection site: Primary patient touchpoint (like hospitals, clinics, and diagnostic centers) involved only with collection of bacterial samples from patients, animals, and the environment, complete with geographic/temporal metadata and supplemented with bacterial species information. Sentinel site: Coordinator of several collection sites, with a robust capacity to perform bacterial identification and antimicrobial susceptibility profiling (in case their satellites lack this). These laboratories, in many cases, can expand capacity to perform downstream activities like DNA extraction before shipping isolates to an RRL/NRL (hub-and-spoke model). RRL: Operates similar to an NRL with a subnational jurisdiction. Performs confirmatory bacterial ID/AST on sentinel site referrals using higher throughput and automated methods and can perform bacterial DNA isolation protocols. If expanded to service a regional demographic, they can potentially transform into low- to medium-throughput WGS centers offering sequencing. NCL/NRL: The largest operation of its kind within a national surveillance setup. Fully equipped to perform large-scale confirmatory phenotypic bacterial/AMR typing on isolates identified and referred by regional satellites (like collection, sentinel, or even RRLs). Due to its size, infrastructure investment, on-hand expertise, and national influence, it is generally the entry point to introduce WGS for national needs. (*b*) To create a coordinated, sustainable flow of samples and data, countries looking to on-board WGS could consider the following network setup models—(left) centralized network: governed by a central, fully kitted, end-to-end genome sequencing center (NRL/NCL) surrounded by satellite collection and sentinel sites; (center) hub-and-spoke network: governed by a central genome sequencing center surrounded by varying degrees of laboratories, some with expandable capacity (shown above in panel *a*) to add growing value to the network; (right) decentralized network: the most advanced form of surveillance where each stakeholder site within a network has some level of WGS capacity on-site. Refer to Supplementary Table 3 for the advantages and potential drawbacks of implementing each of these models. Abbreviations: AMR, antimicrobial resistance; ID/AST, Identification/Antimicrobial Susceptibility Testing; WGS, whole-genome sequencing.

Figure 3 illustrates the possible setup and growth of laboratory networks when gradually incorporating WGS into routine AMR surveillance. Countries implementing WGS with no prior experience in molecular methods might prefer a “centralized” model, where the national reference laboratory fulfills end-to-end genomics. Smaller or newer laboratories often cannot maintain high-throughput instruments and will find it more cost-effective to sequence their isolates at the centrally placed laboratory [6]. Here, a hybrid model, “hub-and-spoke,” would enable peripheral centers to perform preliminary phenotypic assays and sample preparation before transporting DNA to the national laboratory for sequencing. WGS uses DNA as starting input, regardless of organism, sequencing platform, or application. This is advantageous, as peripheral laboratories can ship DNA, often at ambient temperature, to central sites [19]. The most advanced model is completely “decentralized,” with each contributing site within a network fully equipped to autonomously perform some degree of sequencing and data analysis. Cheaper, portable sequencers with integrated compute modules can augment such a decentralized model, providing rapid, real-time WGS processing power [23, 24].

### Procurement

It is evident that a major challenge faced by laboratories in LMICs is a fragmented procurement and support ecosystem in which suppliers work autonomously from manufacturers (Supplementary Table 2). There exists enormous disparity in setup costs for an Illumina MiSeq sequencer between the United Kingdom and 4 low to middle-income sites [4]. To combat similar biases with labware and reagents, purchasing consortia, formed through alliances between several local laboratories, could collectively negotiate pricing with suppliers (especially in regions serviced by a monopoly). Similarly, in a hub-and-spoke setup, the main stakeholder laboratory could negotiate pricing on behalf of any sentinel sites to reduce overall pricing. A further layer of reliability and subsidy can be brought about by the WHO’s involvement as an intermediary through its list of approved suppliers [4]. In our experience, assay costs within individual laboratories could be reduced in 3 ways: (1) exploring multi-vendor solutions—when sample demand is low, utilizing competing vendors across protocols could improve costs over time and prevent monopolistic price locks; (2) ordering reagents and plasticware in bulk (ie, 6–10 months’ worth of requirements); and (3) troubleshooting minor hardware and software faults in-house to shrink periods of inactivity and reduce expensive service call-outs.

### Laboratory Automation

Automating WGS sample processing requires careful time and budget consideration and only becomes cost-effective when sample volumes grow [4]. Pragmatically, a laboratory protocol should only be automated once laboratory staff are fully proficient in the corresponding manual, hand-processed workflow. We believe the ability to fall back upon manual intervention in the event of robot malfunction maintains some level of sample throughput in the interim and prevents overreliance on push-button convenience that can diminish troubleshooting abilities. This holds especially true for regions where hardware service callouts have lengthy turnaround times.

### Personnel Capacity Building

The format and quality of training determines how effectively methods can be delegated to other laboratories within a network, thereby expanding regional expertise. It is impractical for primary trainers to travel to and teach at every far-reaching site within a network. Deploying the Train-the-Trainer system ensures that primaries pass their knowledge onto secondary and tertiary cohorts, thereby accelerating skill sharing and mentoring throughout the consortium. Moreover, we observed that designing experiential workshops that challenge participants with “erroneous” simulations of real-world scenarios vastly enhanced troubleshooting acumen.

As emphasized by Afolayan et al [12], implementing the right data collection and processing infrastructure, alongside building proficiency in pathogen genome analysis can be a formidable barrier to adoption of a surveillance system. The GHRU-AMR initiative has sought to address these barriers and provide viable solutions to a lot of these analysis bottlenecks. We found that, even once obstacles with sequence analyses are overcome, it is fundamentally critical to closely integrate the biologically focused “wet” laboratory workflows with the data-focused “dry” laboratory components. Personnel on either side of this divide (or in many cases, spanning it) must understand the fundamentals of each in order to better optimize processes end-to-end, monitor sequence quality, and modify assay outputs where needed.

### Quality Assurance

External QAs offer ring trials for independent verification of wet-laboratory proficiency and WGS performance [4, 15]. Alongside these, laboratories should also self- or peer-assess their internal protocols using randomized quality exercises that modularly test workflows for all organisms of interest. Internal QA could be run periodically (every 3, 6, or 12 months), and could test either part of, or the entire, workflow. If evaluating specific SOPs—for example, DNA library preparation—the assessor could either provide pure, well-characterized, pre-quantified dsDNA input and evaluate the resulting libraries, select routine sample batches at random for independent evaluation, or introduce blinded, validated controls every few sampling batches.

## Conclusions

On-boarding WGS capabilities can greatly enhance the real-time processing power within regional and national AMR surveillance initiatives, despite the high initial investment in laboratory infrastructure and maintenance. Due to the technology’s demand-driven supply chain, the cost per genome is expected to drop as it becomes more routinely adopted by more regional centers around the world. It is crucial to point out that this encouraging trend can only provide true value when supplemented with robust training, quality monitoring, and local proficiency in performing quality-assured WGS assays. For countries looking to introduce WGS as a surveillance tool, a useful pilot initiative would be to sequence select GLASS priority pathogens (preferably those with genotypically established AMR mechanisms) that can demonstrate the standardization and impact WGS has in tackling AMR [4].

## Supplementary Data

Supplementary materials are available at *Clinical Infectious Diseases* online. Consisting of data provided by the authors to benefit the reader, the posted materials are not copyedited and are the sole responsibility of the authors, so questions or comments should be addressed to the corresponding author.

ciab796_suppl_Supplementary_Material

ciab796_suppl_Supplementary_Table_1

ciab796_suppl_Supplementary_Table_2

## Notes

***Disclaimer.*** The views expressed in this publication are those of the authors and not necessarily those of the National Health Service, the National Institute for Health Research, or the Department of Health.

***Financial support.*** This work was supported by Official Development Assistance (ODA) funding from the National Institute for Health Research [grant number 16_136_111] and the Wellcome Trust grant number 206194.

This research was commissioned by the National Institute for Health Research using Official Development Assistance (ODA) funding. The views expressed in this publication are those of the authors and not necessarily those of the NHS, the National Institute for Health Research or the Department of Health.

***Supplement sponsorship.*** The supplement is sponsored by the UK National Institute for Health Research Global Health Research Unit on Genomic Surveillance of AMR.

***Potential conflicts of interest.*** M. K. reports grants or contracts outside of the submitted work, support for attending meetings and/or travel, and receipt of equipment, materials, drugs, medical writing, and gifts or other services from the National Institute for Health Research (NIHR). G. N. reports support for attending meetings and/or travel from the grant from NIHR, UK, received through their institution. All other authors report no potential conflicts of interest. All authors have submitted the ICMJE Form for Disclosure of Potential Conflicts of Interest. Conflicts that the editors consider relevant to the content of the manuscript have been disclosed.

***Acknowledgments.*** Members of the NIHR Global Health Research Unit on Genomic Surveillance of Antimicrobial Resistance: Khalil Abudahab, Monica Abrudan, Silvia Argimón, Harry Harste, Dawn Muddyman, Ben Taylor, Anthony Underwood, Nicole Wheeler, and Sophia David of the Centre for Genomic Pathogen Surveillance, Big Data Institute, University of Oxford, Old Road Campus, Oxford, United Kingdom, and Wellcome Genome Campus, Hinxton, United Kingdom; Pilar Donado-Godoy, Johan Fabian Bernal, Alejandra Arevalo, and Erik C. D. Osma Castro of the Colombian Integrated Program for Antimicrobial Resistance Surveillance—Coipars, CI Tibaitatá, Corporación Colombiana de Investigación Agropecuaria (AGROSAVIA), Tibaitatá–Mosquera, Cundinamarca, Colombia; K. L. Ravikumar, Varun Shamanna, Vandana Govindan, Akshata Prabhu, D. Sravani, M. R. Shincy, Steffimole Rose, and K. N. Ravishankar of the Central Research Laboratory, Kempegowda Institute of Medical Sciences, Bengaluru, India; Iruka N. Okeke, Ayorinde O. Afolayan, Jolaade J Ajiboye, and Erkison Ewomazino Odih of the Department of Pharmaceutical Microbiology, Faculty of Pharmacy, University of Ibadan, Oyo State, Nigeria; Celia Carlos, June M. Gayeta, and Elmer M. Herrera of the Antimicrobial Resistance Surveillance Reference Laboratory, Research Institute for Tropical Medicine, Muntinlupa, The Philippines; Ali Molloy, alimolloy.com; John Stelling, The Brigham and Women’s Hospital, Boston, Massachusetts, USA; and Carolin Vegvari, Imperial College London, London, United Kingdom.
