# Supplementary material for: Integrating Scalable Genome Sequencing Into Microbiology Laboratories for Routine Antimicrobial Resistance Surveillance
Source: Clin Infect Dis. 2021 Nov 25;73(Suppl 4):S258–66. doi: 10.1093/cid/ciab796 (PMC8634525; doi:10.1093/cid/ciab796)
Supplement: ciab796_suppl_Supplementary_Material [file ciab796_suppl_supplementary_material.docx]

**Supplementary Information**

**Integrating Scalable Genome Sequencing into Microbiology Laboratories for Routine AMR Surveillance**

Mihir Kekre^1^, Stefany Alejandra Arevalo^2^, María Fernanda Valencia^2^, Marietta L. Lagrada^3^, Polle Krystle V. Macaranas^3^, Geetha Nagaraj^4^, Anderson O. Oaikhena^5^, Agnettah M. Olorosa^3^, David M. Aanensen^1^, The NIHR Global Health Research Unit on Genomic Surveillance of Antimicrobial Resistance ^a^

1. Centre for Genomic Pathogen Surveillance, Big Data Institute, University of Oxford, Old Road Campus, Oxford, United Kingdom and Wellcome Genome Campus, Hinxton, UK

2. Colombian Integrated Program for Antimicrobial Resistance Surveillance – Coipars, CI Tibaitatá, Corporación Colombiana de Investigación Agropecuaria (AGROSAVIA), Tibaitatá – Mosquera, Cundinamarca, Colombia

3. Research Institute for Tropical Medicine, Muntinlupa, Philippines

4. Central Research Laboratory, Kempegowda Institute of Medical Sciences, Bengaluru, India

5. Department of Pharmaceutical Microbiology, University of Ibadan, Ibadan, Nigeria

^a^ Members of the NIHR Global Health Research Unit on Genomic Surveillance of Antimicrobial Resistance are listed in the Acknowledgments.

**Supplementary Tables**

**Supplementary Table 1.** Reagents list. (Available in Excel.)

**Supplementary Table 2.** Laboratory implementation experiences in Colombia, India, Nigeria, and the Philippines. (Available in Excel.)

**Supplementary Table 3.** Advantages and Drawbacks of laboratory surveillance setup models.

| **Surveillance Model (Setup)** | **Advantages** | **Potential Drawbacks** |
| --- | --- | --- |
| Centralized | 1. The NRL can handle larger capacity of sample throughput, thereby lowering per-genome costs 2. Central procurement and supply chain for equipment/reagents 3. Sample data managed centrally | Centrally-managed sequencing means the NRL could potentially be a single point of failure to generate surveillance data for the entire country. |
| Hub and Spoke | 1. Takes operational pressure off the central/national facility by upskilling and delegating certain activities to RRLs (for eg. AST typing, DNA extraction and QC), thereby simplifying resourcing and sample transport logistics in the process. | Complexity when managing:   - Decentralized sample tracking - Site-to-site variability in DNA and genome quality - Supply chain variability between sites - Frequency and cost of training and competency assessments for surrounding centers. |
| Decentralized | 1. Autonomy and flexibility for each governing center to manage their own sequencing operation and surveillance individually. | 1. Issues with cross-compatibility, storage and management of datasets when sharing with other surveillance networks outside the region. 2. Substantially larger investment into regional infrastructure and personnel expertise. |

**Supplementary Figures**

**
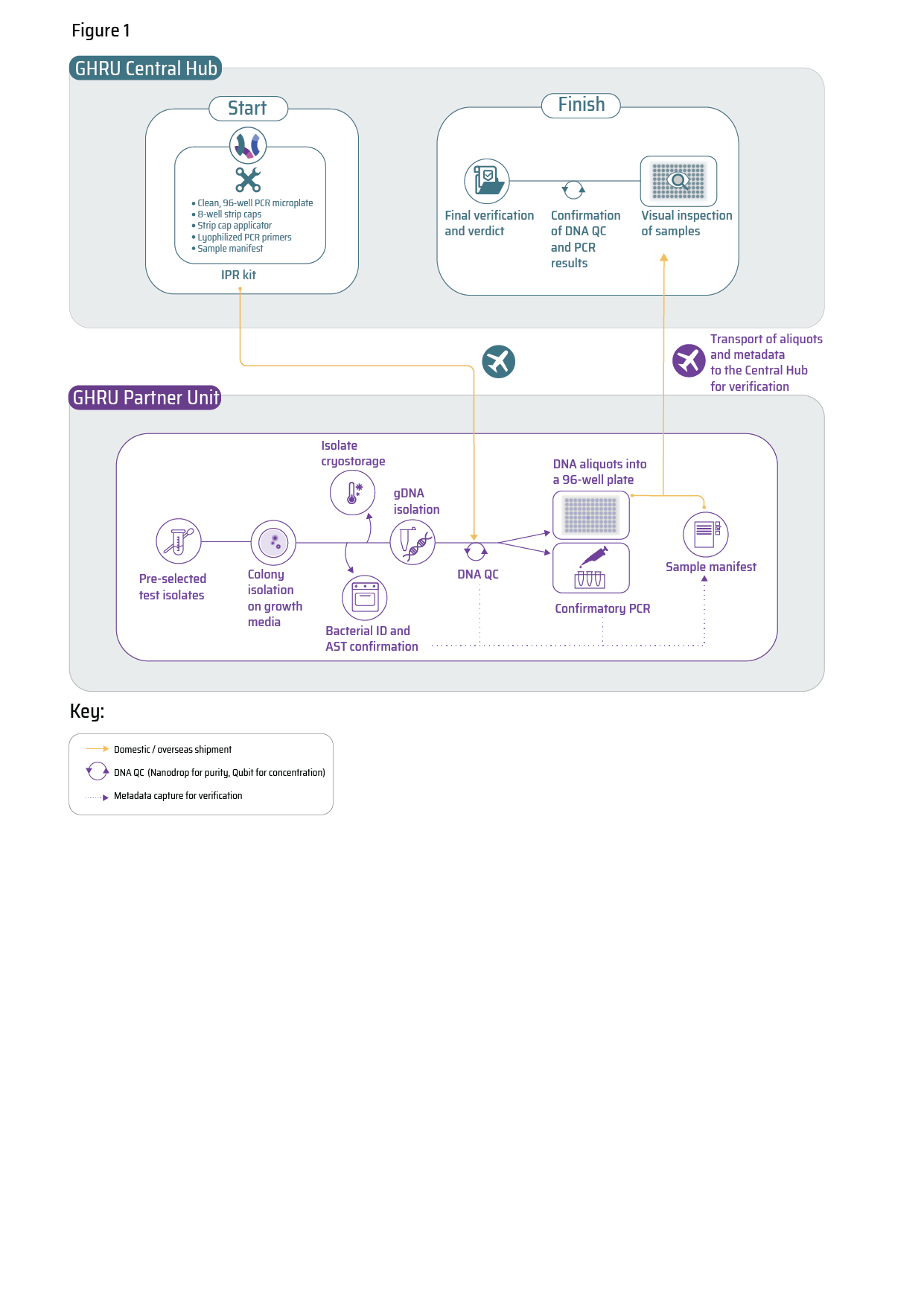
**

**Supplementary Figure 1.**

The Internal Process review (IPR) examined proficiency with: 1) bacterial identification and AST (AMR) characterization, 2) DNA isolation and amplification and 3) secure DNA transport between sites. An IPR kit (to facilitate test sample return) was shipped to each partner unit under evaluation. Pre-selected isolates were cultured and characterized (for ID/AST) with DNA isolated, quantified and amplified using validated primers. The resulting material and results from each site were returned to the Central Hub for verification and final evaluation of competency.


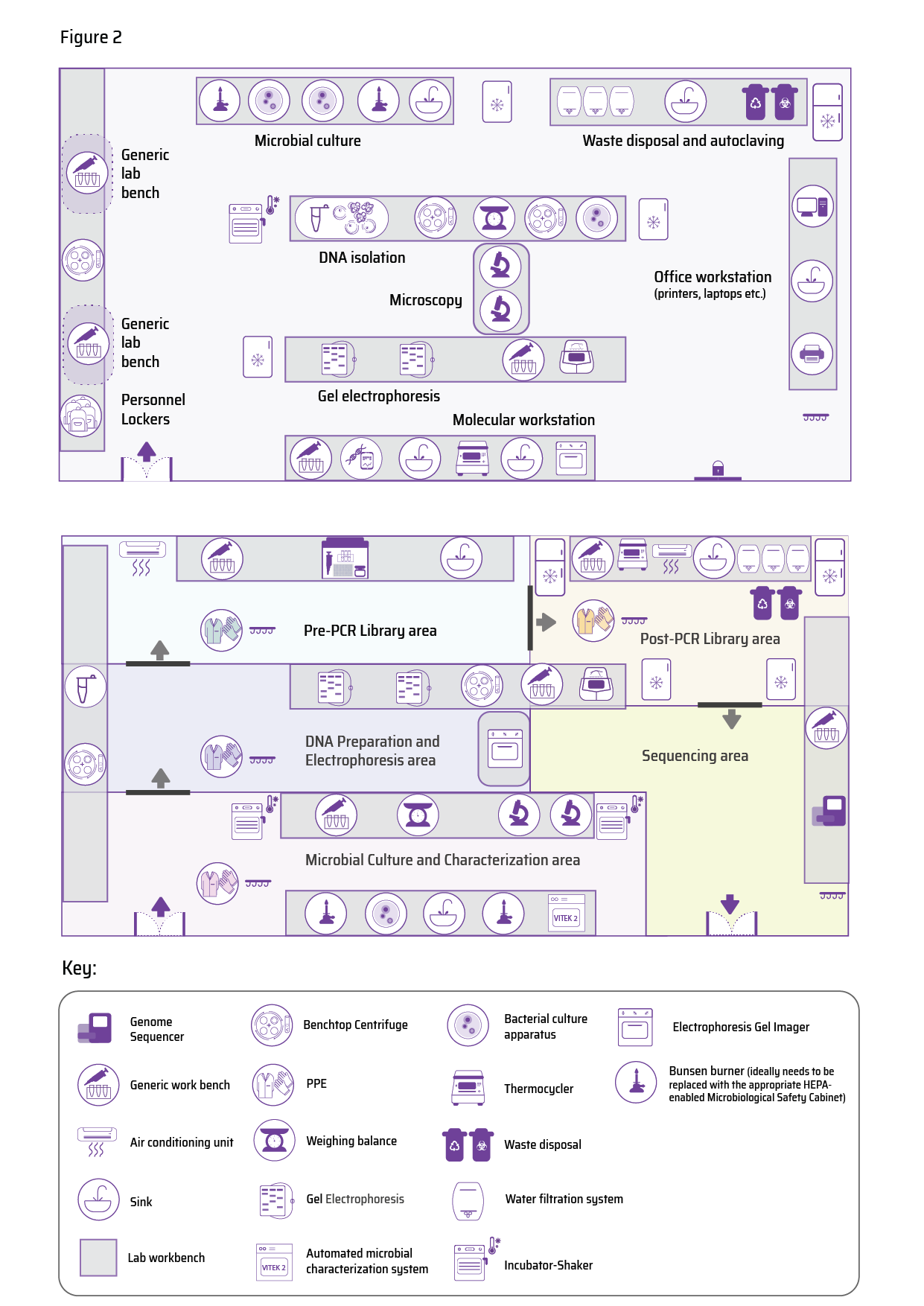


**Supplementary Figure 2.** Laboratory Reconfiguration to augment existing microbial processing with WGS and genomics.

“BEFORE” (top) – An AMR laboratory layout with basic molecular capacity. “AFTER” (bottom) – The same AMR laboratory layout restructured and supplemented with genome sequencing capabilities downstream to microbiology.
